# Plasma Metabolomic Profiling of Patients with Diabetes-Associated Cognitive Decline

**DOI:** 10.1371/journal.pone.0126952

**Published:** 2015-05-14

**Authors:** Lin Zhang, Meng Li, Libin Zhan, Xiaoguang Lu, Lina Liang, Benli Su, Hua Sui, Zhengnan Gao, Yuzhong Li, Ying Liu, Benhui Wu, Qigui Liu

**Affiliations:** 1 Academy of Integrative Medicine, Dalian Medical University, Dalian, Liaoning, China; 2 Department of Traditional Chinese Medicine, the Second Affiliated Hospital, Dalian Medical University, Dalian, Liaoning, China; 3 Department of Emergency Medicine, Zhongshan Hospital, Dalian University, Dalian, Liaoning, China; 4 Department of endocrinology, the Second Affiliated Hospital, Dalian Medical University, Dalian, Liaoning, China; 5 Department of endocrinology, Dalian Municipal Central Hospital Affillated of Dalian Medical University, Dalian, Liaoning, China; 6 Examination Department, the Second Affiliated Hospital, Dalian Medical University, Dalian, Liaoning, China; 7 Medical Examination Center, the Second Affiliated Hospital, Dalian Medical University, Dalian, Liaoning, China; 8 Public Health, Dalian Medical University, Dalian, Liaoning, China; Mayo Clinic, UNITED STATES

## Abstract

Diabetes related cognitive dysfunction (DACD), one of the chronic complications of diabetes, seriously affect the quality of life in patients and increase family burden. Although the initial stage of DACD can lead to metabolic alterations or potential pathological changes, DACD is difficult to diagnose accurately. Moreover, the details of the molecular mechanism of DACD remain somewhat elusive. To understand the pathophysiological changes that underpin the development and progression of DACD, we carried out a global analysis of metabolic alterations in response to DACD. The metabolic alterations associated with DACD were first investigated in humans, using plasma metabonomics based on high-performance liquid chromatography coupled with quadrupole time-of-flight tandem mass spectrometry and multivariate statistical analysis. The related pathway of each metabolite of interest was searched in database online. The network diagrams were established KEGGSOAP software package. Receiver operating characteristic (ROC) analysis was used to evaluate diagnostic accuracy of metabolites. This is the first report of reliable biomarkers of DACD, which were identified using an integrated strategy. The identified biomarkers give new insights into the pathophysiological changes and molecular mechanisms of DACD. The disorders of sphingolipids metabolism, bile acids metabolism, and uric acid metabolism pathway were found in T2DM and DACD. On the other hand, differentially expressed plasma metabolites offer unique metabolic signatures for T2DM and DACD patients. These are potential biomarkers for disease monitoring and personalized medication complementary to the existing clinical modalities.

## Introduction

Type 2 diabetes (T2DM) is a chronic metabolic disorder characterized by hyperglycaemia resulting from insulin resistance and insufficiency [1]. Diabetes has been linked to a 50% increased risk of dementia [2, 3], and disease onset in midlife has been associated with an increased long—term risk of dementia [2]. With an occurrence of T2DM in 12–25% of people aged 65 and up [4, 5], approximately one in ten to one in fifteen dementia cases worldwide can be attributed to T2DM (population attributable risk). If pre-diabetes is also taken into account, these estimates increase to one in seven to one in ten dementia cases [4]. Efforts to understand the pathophysiological changes that underpin the development and progression of diabetes—related cognitive dysfunction (DACD) are of vital importance in the development of treatments to reverse or prevent these cognitive complications.

Metabolomics, the global assessment of endogenous small molecule metabolites within a biological system [6], provides a powerful platform for identifying biomarkers and understanding biochemical pathways to improve diagnosis, prognosis, and treatment of disease [7, 8]. It has been successfully utilized in diabetes for metabolomic profiling using either human or animal model of diabetes mellitus (DM) biofluids (obese Zucker rat, db/db mouse, ddY-H mouse and streptozotocin (STZ) rat) [9–15]. Data from metabolomic analyses of DM indicate that alterations in sugar metabolites, amino acids, and choline-containing phospholipids, are associated early on with a higher risk of T2DM [16–18].

However, there have been no reports of metabolomic studies on DACD. Consequently, this study was designed to provide a comprehensive evaluation and comparison of the metabolome of patients with DACD and T2DM. HPLC-Q-TOF-MS was used in combination with pattern recognition methods and pathway analysis, to look for diversity in the metabolic phenotype, explore the diagnostic possibilities, define new potential biomarkers, and generate a better understanding of the pathophysiology.

## Materials and Methods

### Materials

The metabolite standards used in this study were purchased from Toronto Research Chemicals Inc. (Toronto, Canada). The organic solvents and internal standards used in this study were purchased from Wako Pure Chemical Industries, Ltd (Osaka, Japan).

### Study Subjects and Plasma Collection

Plasma samples from 24 diabetic patients, 24 patients with DACD, and 24 healthy controls were collected from the Endocrine wards and Health examination center of the Second Hospital of Dalian Medical University (Dalian, China) between September, 2011 and September, 2012 without hypertension, renal or liver dysfunction. The study protocol was in accordance with the Helsinki declaration and approved by the ethics committee of Dalian Medical University with written informed consent from all participants. To focus on the objectives of this study and exclude the effects of age, gender, and obesity, a total of 200 age-, gender-, and body mass index (BMI)-matched persons were selected and divided into three groups (health control, T2DM, DACD, *p*<0.05) according to WHO criteria of diagnosis of diabetes, MMSE and MoCA. Among the T2DM and DACD groups, patients were all newly diagnosed thus without any treatments. The demographic and clinical chemistry characteristics of enrolled subjects are shown in Table 1. The subjects fasted for at least 12 hours before blood draw. The blood sample was collected using heparin (10 UI/mL) as anticoagulantin graduated ice-cold polypropylene tubes. Plasma samples were immediately prepared by centrifugation (3000 rpm for10 min) and stored at −80°C until use for metabonomics analysis.

**Table 1:** Demographic and Clinical Chemistry Characteristics of T2DM, and DACD and healthy control.

	T2DM	DACD	healthy controls
no. of subjects	24	24	24
sex(M/F)	12/12	12/12	12/12
age (median/range)	58/46-69	57/46-70	56/47-68
BMI (median/range, kg/m^2^)	26.46/23.05–33.38	27.47/22.30–30.51	25.46/22.61–32.85
SBP(median/range, mmHg)	140/100-194	148/116-190*	126/98-147
DBP(median/range, mmHg)	82/60-100	80/50-110	79/51-95
FPG(median/range, mmol/L)	7.94/4.65–16.16**	9.18/5.25–21.49**	5.52/5.04–6.21
2hPG(median/range, mmol/L)	13.59/7.1–22.95**	13.29/7.54–29.74**	5.87/5.46–7.36
CH(median/range, mmol/L)	5.24/3.92–7.85	4.73/2.98–7.16*	5.17/3.52–6.46
TG(median/range, mmol/L)	1.93/0.84–8.3**	1.83/0.72–4.58	1.22/0.56–2.48
HDL-C(median/range, mmol/L)	1.04/0.66–1.53	1.07/0.68–1.77*	1.24/0.77–2.01
LDL-C(median/range, mmol/L)	3.07/1.81–4.54	2.79/1.27–4.51	3.28/1.89–4.65
FINS (median/range, MIU/L)	7.3/4.14–25.08**	7.95/5.27–28.65**	7.54/6.06–12.74

*P<0.05

**P<0.01 compared with healthy control

### Sample Preparation and Pretreatment

Prior to LC/MS analysis, plasma samples were thawed at room temperature for 15 min, vortexed vigorously for 5s, and then 300 μL of HPLC grade methanol (Fisher) was added to 100 μL of the plasma samples and vortexed vigorously for another 30s. The sample mixture was allowed to stand for 20 min at 4°C and centrifuged at 12,000 rpm for 15 min at 4°C. The supernatant (200 μL) was transferred to afresh tube then evaporated to dryness by nitrogen blowing, then 200 μL of 80% methanol were added and vortex-mixed. This resolublized solution was recentrifuged once again, and the supernatant was then transferred to a high performance liquid chromatograph (HPLC) autosampler injection vial for LC/MS analysis. To ensure the stability and repeatability of the HPLC-Q-TOF systems, pooled quality control (QC) samples were prepared from 10 μl of each sample and staggered with the other samples (after every ten samples).

### HPLC-QTOF/MS analysis

HPLC-QTOF/MS analysis was performed on 5 μL aliquot of the pretreated plasma samples using a C18 (2.1 mm×100 mm×1.8 μm) column (Agilent Technologies, Santa Clara, CA, USA) held at 40°C using an Agilent 1260 Infinity LC System (Agilent Technologies). The metabolites were eluted with a gradient of 2% B for 0−2min, 2−95% B for 2−17 min, and kept 95% B for 17−19 min. For positive ion mode (ES+) where A = water with 0.1% formic acid, B = acetonitrile with 0.1% formic acid, while A = water and B = acetonitrile for negative ion mode (ES-). The flow rate was 0.4 mL/min, and samples were maintained at 4°C during the analysis.

Mass spectrometry was performed using an Agilent 6530 UHD and Accurate-Mass Q-TOF (Agilent Technologies) equipped with an electrospray ionization source operating in either positive or negative ion mode. The source temperature was set at 100°C with a cone gas flow rate of 50 L/h. The desolvation gas temperature was 350°C with a flow rate of 600 L/h. The capillary voltage and the cone voltage were set to 4 kV and 35 V, respectively. Centroid data were collected from 50 to 1000 m/z with a scan time of 0.03 s, and an interscan delay of 0.02 s. All analyses were acquired using the lock spray feature to ensure accuracy and reproducibility. Leucine-enkephalin was used as the lock mass (m/z 556.2771 in ES+ and 554.2615 in ES-).

### Data Preprocessing and Annotation

The raw HPLC-QTOF/MS ESI data were converted tomz format data by MassHunter(Agilent), and the files then imported to the XCMS package (R program) for preprocessing, including nonlinear retention time (RT) alignment, matched filtration, peak detection, and peak matching [19].

Finally, the output data was manually searched and edited in EXCEL2007 software, including the elimination of impurity peaks and duplicate identifications. The final results were changed to 2 D data matrix, including variance (Rt/mz), observed quantity and peak intensities.

### Statistical Analysis

The two data sets resulting from HPLC-QTOF/MS ES+ and ES- were mean centered, unit variance scaled and combined before uni- and multivariate statistical analysis in SIMCA-p 11.0 Software package (Umetrics AB, Umea, Sweden), SPSS (v19, IBM, New York, NY), and Matlab. Both the unsupervised method (principal component analysis, PCA) and the supervised method (Orthogonal partial least-squares discriminantanalysis, OPLS-DA) were employed to reveal the global metabolic changes between T2DM and DACD, T2DM and healthy controls, and DACD and healthy controls using SIMCA-p 11.0 (Umetrics AB, Umea, Sweden). The corresponding variable importance in the projection (VIP values) was calculated in the OPLS-DA model as well. A validation plot was used to assess the validity of the OPLS-DA model by comparing the goodness of fit of the OPLS-DA models with the goodness of fit of 100 Y-permutated models. On the basis of a VIP threshold of 1, from the 7-fold cross-validated OPLS-DA model, a number of metabolites leading to the difference in the metabolic profiles of diseased individuals and healthy controls were obtained. The differential metabolites were uncovered based on p value of bilateral asymptotic significance (Mann—Whitney U test, p < 0.05). The corresponding fold change shows how these selected differential metabolites varied between the T2DM and healthy control groups, DACD and healthy control groups and T2DM and DACD.

The goodness-of-fit parameters for the OPLS model, R2X, R2Yand Q2Y, were calculated which varied from 0 to 1. R2X and R2Y represent the fraction of the variance of the x and y variable explained by the model, while Q2Y suggests the predictive performance of the model. For internal validation of the OPLS models, a permutation test (100 permutations) was performed. This evaluated whether the OPLS models, built with the groups, was significantly better than any other OPLS model obtained by randomly permuting the original group attributes.

Statistical analysis was performed using the R platform [20], with the exception of PCA and OPLS-DA, which were carried out on SIMCA-p. Two binary logistic regression (BLR) models were established for biomarkers discovery and discrimination of T2DM patients, DACD patients and healthy controls (SPSS, version 11.5). Two thirds subjects were used as the training set to build BLR model, and the remaining one third subjects were used as test set for the validation of the model. Area under the curve (AUC) of receiver operating characteristic (ROC) curve analysis was applied to evaluate the diagnostic capacity of individual metabolites. The resultso f ROC curves and final error rates of training, test, and validation sets were on the basis of these outcomes, which should guarantee the reliability of potential biomarkers for independent validation. Heat map employing MeV 4.7.4 was carried out to project the metabolic regulations of the differential metabolites.

### Identification of Plasma Biomarkers

For the identification of potential biomarkers, some available biochemical databases, such as HMDB (http://www.hmdb.ca/), KEGG (http://www.genome.jp/kegg/), METLIN (http://metlin.scripps.edu/), LIPIDMAPS (http://www.lipidmaps.org/) and Chemspider (http://www.chemspider.com) were used by comparing the accurate mass within 10 ppm, fragments information and MS/MS data obtained from HPLC-Q-TOF/MS. The list of metabolites after database matching is shown in S1 Table. Moreover, nine potential biomarkers among them were further identified by comparing with reference standards. The interactions between metabolites of interest were analyzed by the KEGGSOAP software package, and the network diagrams in which parameter were set within 5-step reactions was established by Cytoscape.

## Results

### Metabolic Profiles ofT2DM, DACD and Healthy Controls

**Fig 1:**
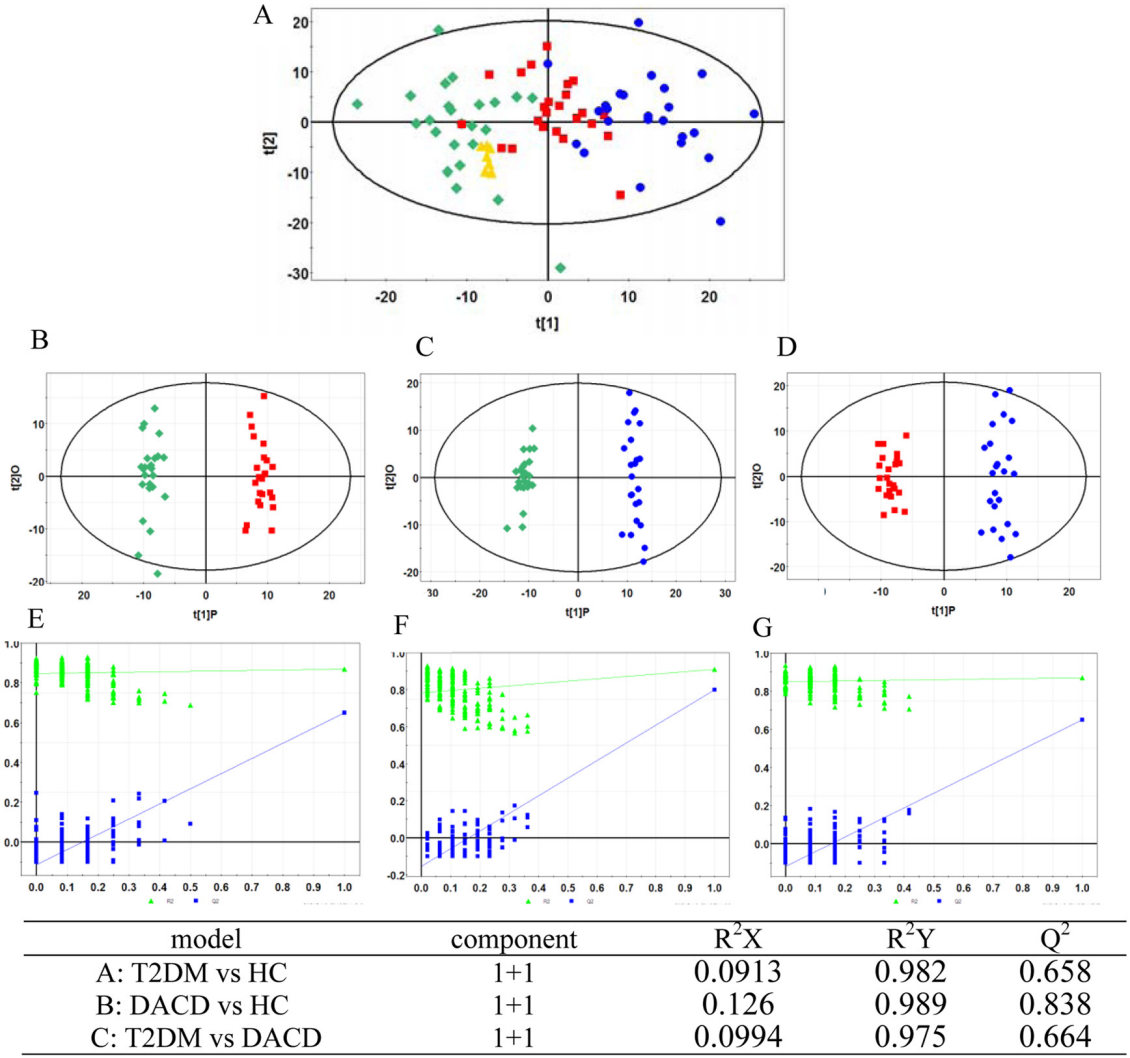
PCA model and OPLS-DA models with corresponding values of R2X, R2Y, and Q2. (A) PCA score plot of healthy controls (green diamond), T2DM patients (red square), DACD patients (blue circle) and QC samples (yellow triangle); (B) OPLS-DA score plot of healthy controls (green diamond) vs T2DM patients (red square); (C) OPLS-DA score plot of healthy controls (green diamond) vs DACD patients (blue circle); (D) OPLS-DA score plot of T2DM patients (red square) vs DACD patients (blue circle); (E, F, G) Validation plot obtained from 100 tests, respectively.

The typical HPLC-QTOF/MS chromatograms are shown in S1 Fig. The final data table contained 1639 variables (chromatographic peaks). A principal component analysis (PCA) was first performed to show a trend of intergroup separation on the scores plot (Fig 1A), in which T2DM and DACD patients were clearly separated from healthy controls. This method also enabled detection and exclusion of any outliers, defined as observations located outside the 95% confidence region of the model. The OPLS-DA models indicate clear separations between T2DM (red dots) and health control (green dots) groups (R2X = 0.09, R2Y = 0.98, Q2 = 0.66, Fig 1B), DACD (blue dots) and health control (green dots) groups (R2X = 0.13, R2Y = 0.99, Q2 = 0.84, Fig 1C), T2DM (red dots) and DACD (blue dots) groups (R2X = 0.10, R2Y = 0.98, Q2 = 0.66, Fig 1D).

### Discovery and Identification of Metabolic Biomarkers

The OPLS-DA S-plots were shown in S3 Fig, and potential markers were extracted based on their contribution to the variations and correlation within the dataset. A total of 97 significantly altered plasma features with a VIP threshold (VIP > 1) from the aforementioned OPLS-DA model as well as the FDR values based on two-sided p-values calculated from nonparametric Kruskal-Wallis rank sum test (FDR<0.05) were selected and their variations are summarized in S1 Table. These 97 metabolites were identified through MS/MS and online databases: 56 metabolites for T2DM vs health control, 67 metabolites for DACD vs health control and 33metabolites for T2DM vs DACD. Nine of them, including glycerophosphocholine, phytosphingosine, glycocholic acid, sphingosine-1-phosphate, sphinganine-phosphate, pyroglutamic acid, hypoxanthine, cholic acid and linoleic acid were additionally verified by external reference standards (S2 Table and the identification of glycocholic acid was shown in S2 Fig). The % RSD of these 97 metabolites from plasma QC samples varied from 2.4 to 19.3% with a median of 7.2%, which indicated the robustness of our metabolic profiling platform, and this robustness could be suggested by the PCA scores plot comprising T2DM, DACD, healthy control, and the QCs as well.

### Metabolic profile of T2DM and DACD compared with healthy controls

**Fig 2:**
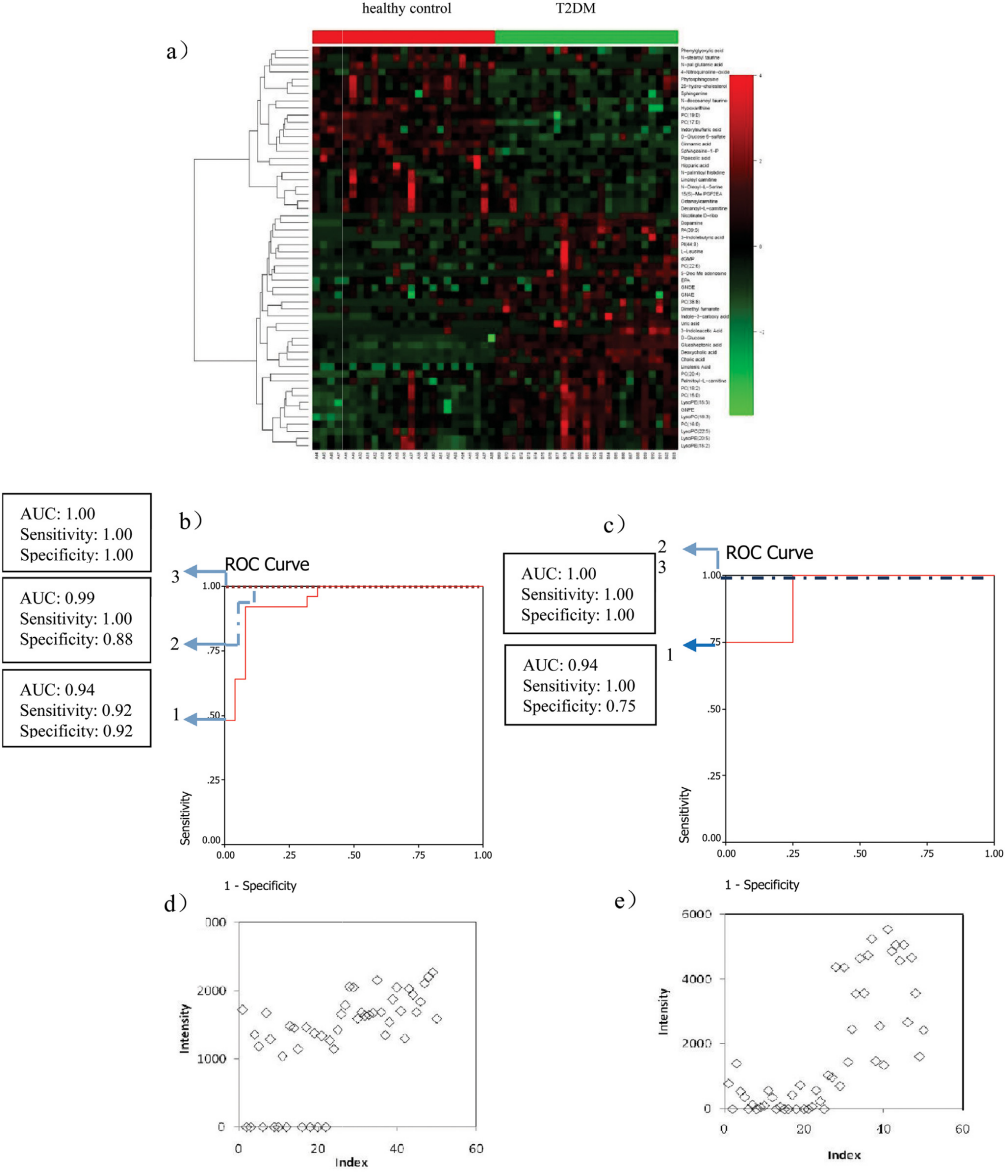
Comparison of metabolomic profiles from T2DM patients vs healthy controls. (a) Heat-map of fold change of 56 differential metabolites; (b) Discrimination of T2DM in the training set using Linolenic Acid (1), deoxycholic acid (2), and the combination of them (3). (c) Discrimination of T2DM in the test set using Linolenic Acid (1), deoxycholic acid (2), and the combination of them (3); (d) Scatter plot of Linolenic Acid; (e) Scatter plot of deoxycholic acid.

From the 56 metabolites differentiating T2DM patients from the healthy control group (heat-map displayed in Fig 2a), 8 metabolites (deoxycholic acid, cholic acid, 25-hydroxy-cholesterol, cinnamic acid, 3-Indolebutyric acid, uric acid, PA(39:5), Linolenic Acid) were selected by two steps, first, ranking the separating capacity (in descending order) of the annotated metabolites by their VIP, *p*-value and absolute fold change, respectively, and three lists were built. Secondly, the selected metabolites fall in the first 50% (top 28) of all the lists. Then, Two-thirds of the patients in each group were selected as a training set, with the remaining patients forming the test set. Linolenic acid and deoxycholic acid which were significantly contributed to the diagnosis of T2DM, were selected by the binary logistic regression analysis. Their combination can correctly predict 100% patients with the corresponding AUC equal to 1.00 in the training set and can correctly predict 100% patients with the corresponding AUC equal to 1.00 in the test set (Fig 2b and 2c). The scatter plots of linolenic acid and deoxycholic acid are shown in Fig 2d and 2e.

**Fig 3:**
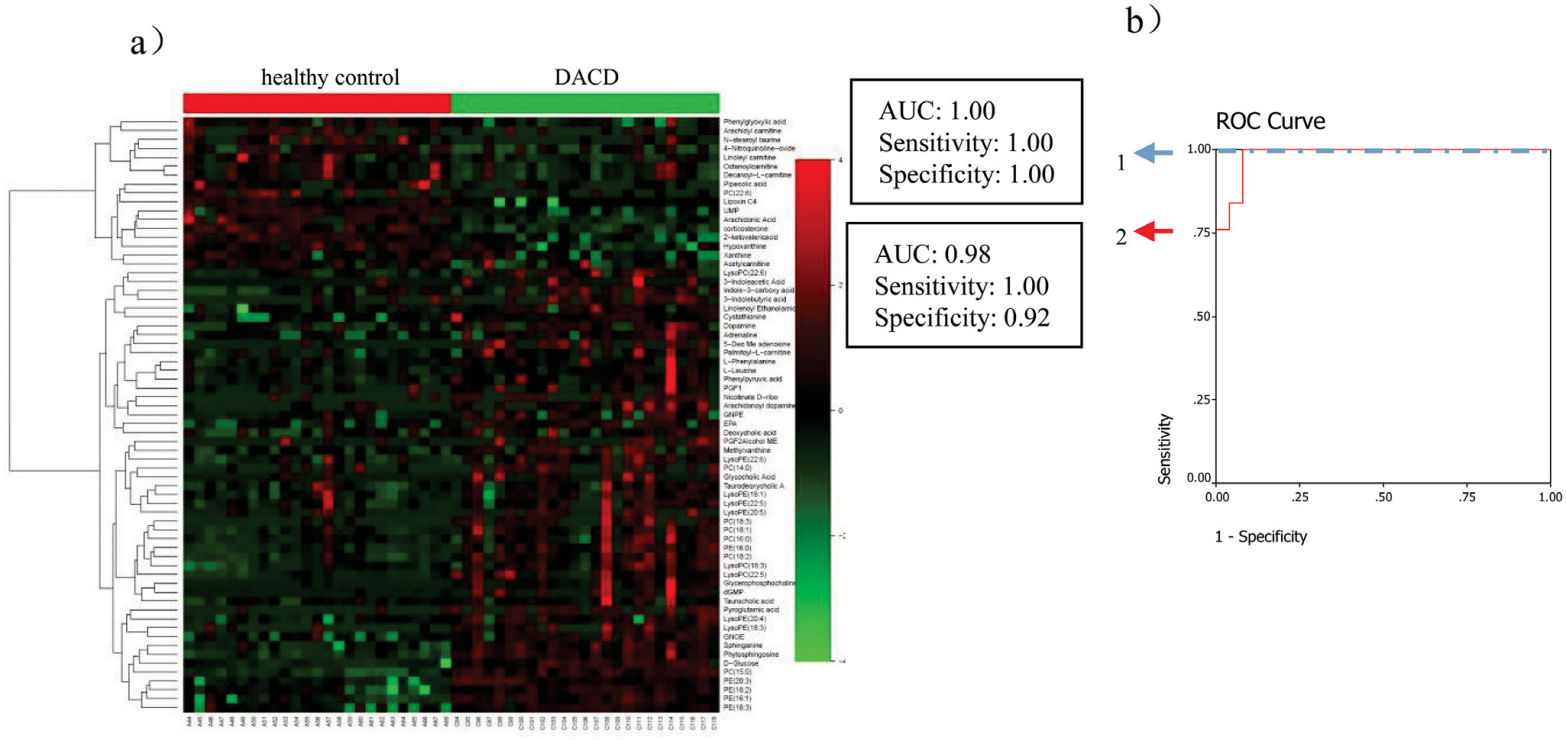
Comparison of metabolomic profiles from DACD patients vs healthy controls. (a) Heat-map of fold change of 66 differential metabolites; (b) Discrimination of DACD in the training set (1) and in the test set (2) using phytosphingosine.

Among the 67 metabolites differentiating DACD patients from the healthy control group (heat-map as shown in Fig 3a), 10 metabolites (Glycerophosphocholine, 5'-Deoxy-5'-(methylthio)adenosine, PC(18:3), Pyroglutamic acid, LysoPE(18:3), 3-Methylxanthine/7-Methylxanthine/1-Methylxanthine, Phytosphingosine, LysoPC(22:5), PC(14:0), 2-keto valeric acid) were selected as mentioned above. Only phytosphingosine which was significantly contributed to the diagnosis of DACD, was selected by the binary logistic regression analysis. Phytosphingosine can correctly predict 100% patients with the corresponding AUC equal to 1.00 in the training set and can correctly predict 92% patients with the corresponding AUC equal to 0.98 in the test set (Fig 3b).

### Metabolite Markers Identified between T2DM and DACD, for Classification of the Two Conditions

**Fig 4:**
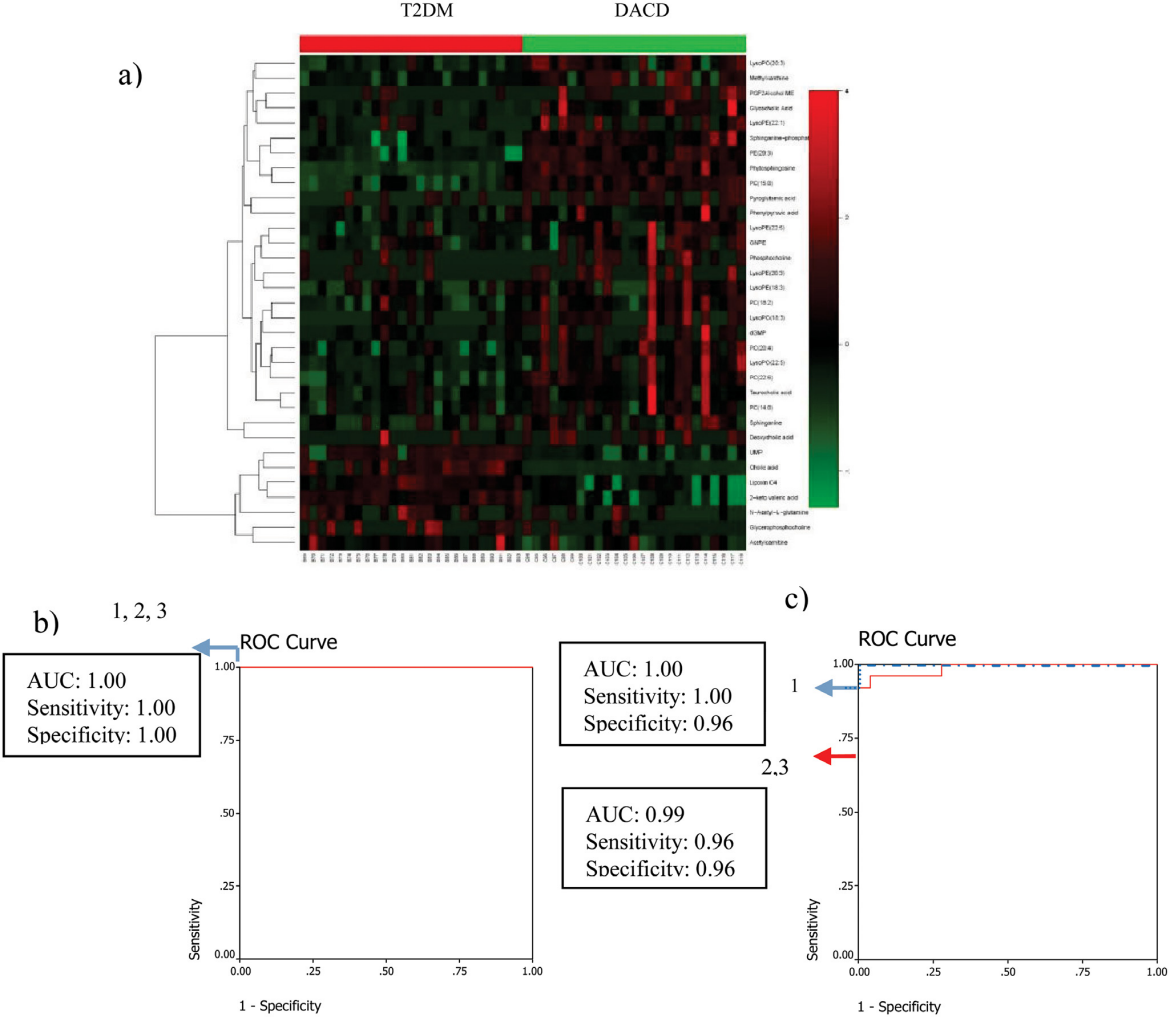
Comparison of metabolomic profiles from T2DM vs DACD patients. (a) Heat-map of fold change of 33 differential metabolites; (b) Discrimination T2DM from DACD in the training set using phytosphingosine (1), sphinganine-phosphate (2), and the combination of them (3). (c) Discrimination T2DM from DACD in the test set using phytosphingosine (1), sphinganine-phosphate (2), and the combination of them (3).

Similarly, 9 metabolites (Phytosphingosine, PC(22:6), PE(20:3), 2-keto valericacid, Sphinganine-phosphate, Cholic acid, Deoxycholic acid, 3-Methylxanthine/7-Methylxanthine/1-Methylxanthine, Uridine monophosphate) were selected from 33 metabolites (heat-map displayed in Fig 4a) between T2DM and DACD patients. Phytosphingosine and sphinganine-phosphate which were significantly contributed to distinguish DACD from T2DM, were selected by the binary logistic regression analysis. Phytosphingosine and sphinganine-phosphate can correctly predict 100% patients with the corresponding AUC equal to 1.00 in the training set (Fig 4b) and can correctly predict 99% patients with the corresponding AUC equal to 0.99 in the test set (Fig 4c).

## Discussion

Emerging evidence suggests that diabetes increases the incidence of dementia to develop to DACD [21–26]. DACD is one of chronic complications in diabetic patients, which was diagnosed by using TCD, rheoencephalogram, brain CT scan, nuclear magnetic resonance spectrum, MoCA and MMSE for cognitive dysfunction, and clinical manifestation and serum indexes for diabetes mellitus. However, none of these methods is suitable for the clinically exclusive diagnosis of DACD at an early stage. Therefore, a non-invasive method and sufficiently sensitive and specific biomarkers are urgently needed for assessing the presence and progression of DACD. Plasma metabonomics based on HPLC/Q-TOF MS has been of great value in the discovery of biomarkers and the elucidation of the pathogenesis of various diseases [27–29]. There were a lot of researches on the metabonomics study of T2DM, and most of differential metabolites identified in our studies were reported before [30]. However, there has been no report on plasma metabonomics study of DACD patients. Therefore, this unbiased global plasma metabonomics study based on HPLC/Q-TOF MS coupled with multivariate statistical analysis is the first to identify potential biomarkers and unravel the molecular mechanisms of DACD.

To gain insight into the metabolic mechanism of DACD and to provide accurate treatment informations of DACD, altered metabolic pathway and the network relationship associated with DACD has been further investigated. Therefore, we developed a research approach consisting of the following steps. First, the related pathway of each metabolite of interest was searched in the database. Second, the interactions between metabolites of interest were analyzed by the KEGGSOAP software package. Third, the network diagrams in which parameter were set within 5-step reactions was established by Cytoscape. Fourth, the metabolic mechanism was presumed by network analysis.

### Sphingolipids metabolism

**Fig 5:**
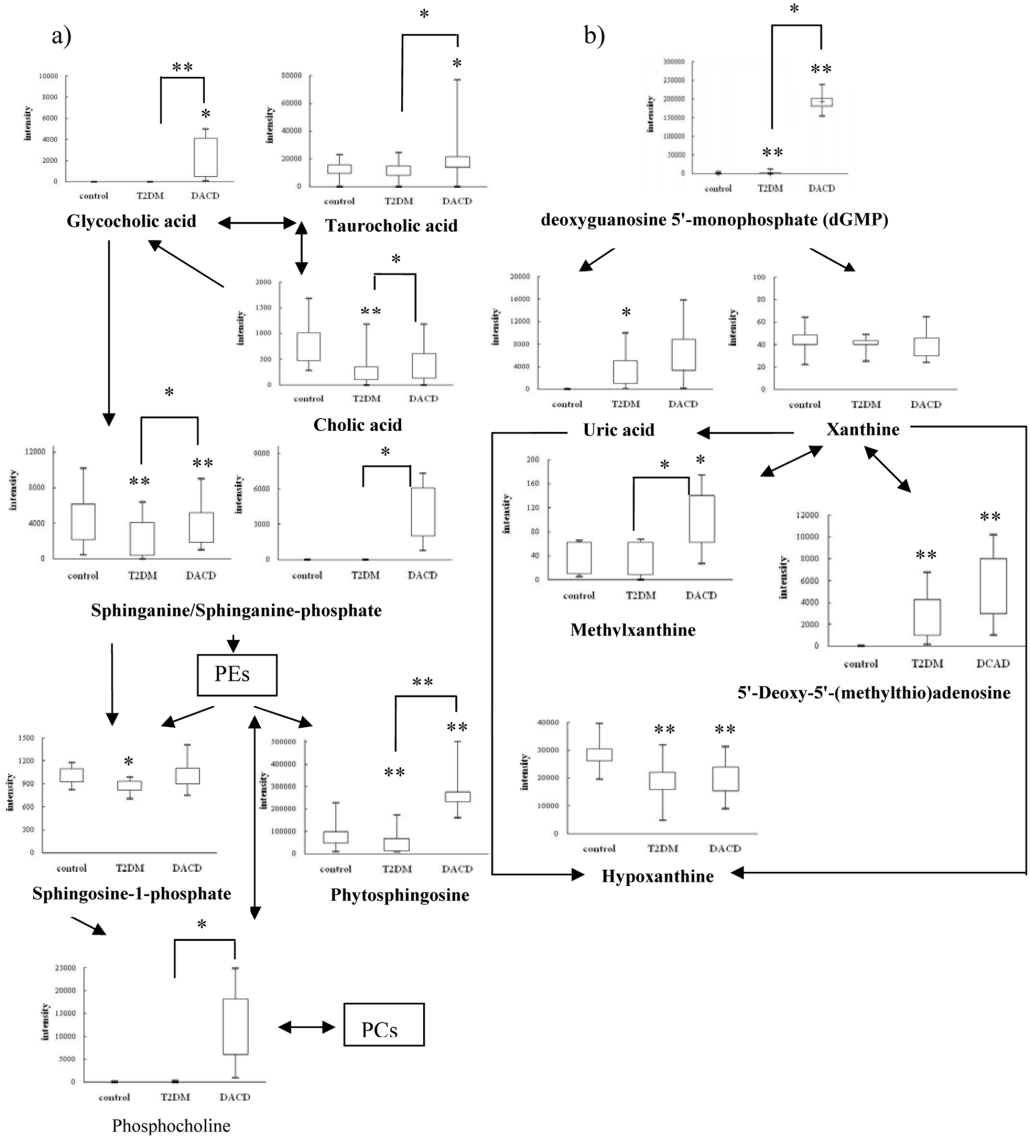
Metabolic network of the significantly changed metabolites within 5 steps by cytoscape software package. The normalized contents are shown under the chemical name. All the p values were calculated using Student t test., *p< 0.05, **p< 0.01.

Sphingolipids are essential lipids consisting of a sphingoid backbone that is N-acylated with various fatty acids to form many ceramide species, which can have hundreds of distinct head groups [31]. Sphingolipids have a rapid turnover and their levels are controlled by the balance between synthesis and degradation in multiple compartments [32]. Their metabolism could affect cell growth, differentiation and behavior. Previous studies have shown that sphingolipids contribute to insulin resistance, diabetes, Alzheimer’s disease and other diseases [33–35]. Interestingly, we saw a significant decrease in levels of phytosphingosine, sphinganine and sphingosine-1-phosphate in T2DM patients, however, levels of phytosphingosine and sphinganine were elevated in DACD patients (Fig 5a), which is consistent with the reported changing tendency of sphingolipids metabolism in the plasma of T2DM patients [36], and there was no report about that in the plasma or other body fluid of DACD patients. But the sphingolipids were found to be elevated in the plasma of patients with mild cognitive impairment and alzheimer's disease, which possessed some correlation with our results [29].

### Bile acids metabolism

Bile acids have long been known to be essential in dietary lipid absorption and cholesterol catabolism, and have a role in regulating thyroid hormone signaling and energy homeostasis [37]. In healthy individuals, only small quantities of bile acids are found in peripheral circulation and plasma. However, in patients with hepatobiliary and intestinal disease, disturbances of synthesis, metabolism, and clearance by the liver and absorption by the intestine affect the concentration and profile of bile acids in various compartments (plasma, liver, gallbladder, urine and feces) [38]. In our study, increased cholic acid level was observed in T2DM patients, similarly with that in the urine of streptozotocin-induced diabetic rats [39, 40]. Increased levels of glycocholic acid and taurocholic acid were observed in DACD patient, which was consistent with that in the plasma of MCI and AD patients [40].

### Uric acid metabolism

Uric acid produced by hypoxanthine and xanthine under the catalysis of xanthine oxidase, is the end product of purine metabolism in humans. Although uric acid is a potent antioxidant in the extracellular environment, when uric acid enters cells via specific organic anion transporters, it induces an oxidative burst in vascular smooth muscle cells, endothelial cells, adipocytes, islet cells, and hepatocytes, which may increase the risk for metabolic syndrome [41–44]. In our study, decreased hypoxanthine and xanthine levels, and increased uric acid levels were observed in T2DM and DACD patients as shown in Fig 5b, although, eliminating uric acid in DACD and xanthine in T2DM after FDR corrections, which was consistent with that in T2DM and vascular dementia patients, as reported previously [45, 46]. Therefore, an elevated serum uric acid was reported to be one of the best independent predictors of diabetes and commonly precedes the development of diabetes [44]. Similarly, an increase in xanthine oxidase activity has also been observed in the plasma of T2DM patients, which may be the reason of the upregulation of uric acid and downregulation of hypoxanthine and xanthine [47]. Other research groups have also shown that increased uric acid levels in plasma result in an elevated risk of cognitive impairment [48, 49]. Furthermore, upregulation of deoxyguanosine 5'-monophosphate (dGMP) and 5'-Deoxy-5'-(methylthio)adenosine in T2DM and DACD patients, upregulation of methylxanthine in DACD patients were observed, which has not been reported before, and may be the direct or indirect reason of uric acid elevated. Therefore, in clinic, we not only need to control fasting blood-glucose, blood pressure, blood lipid levels and obesity, but also should pay attention to the risk factor of hyperuricemia and give relative intervention measures in a timely fashion.

There were some limitations to our studies. Clearly our observations need to be replicated and verified in other larger cohorts, and more informations should be got from muti-methods for example HILIC column, GC-MS, NMR and so on. This issue is currently being examined in follow-up studies. In particular it will be important to evaluate the additional value of metabolomic measurements compared to established clinical pathology data if the metabolites observed here associated with DACD are to be considered as valuable biomarkers in the future. We are further investigating the expression of proteins involved in the identified pathways in T2DM and DACD patients to further validate these potential biomarkers.[21][26]

## Supporting Information

S1 Fig The HPLC-MS TICs of plasma samples from three groups as technical replicates. (TIF)

S2 Fig The [M+H]^+^, [M-H]^-^ and MS/MS spectrum of glycocholic acid. (TIF)

S3 Fig The results of S-plots of OPLS-DA models. (A) T2DM vs Health control. (B) DACD vs Health control. (C) DACD vs T2DM.
(TIF)

S1 Table Statistical analysis result of differential metabolites. (PDF)

S2 Table The informations of reference standards. (PDF)

S3 Table The detailed results from the pathway analysis. (PDF)
