# Supplementary figures and images for: Plasma Metabolomic Profiling of Patients with Diabetes-Associated Cognitive Decline
Source: PLoS One. 2015 May 14;10(5):e0126952. doi: 10.1371/journal.pone.0126952 (PMC4431856; doi:10.1371/journal.pone.0126952)

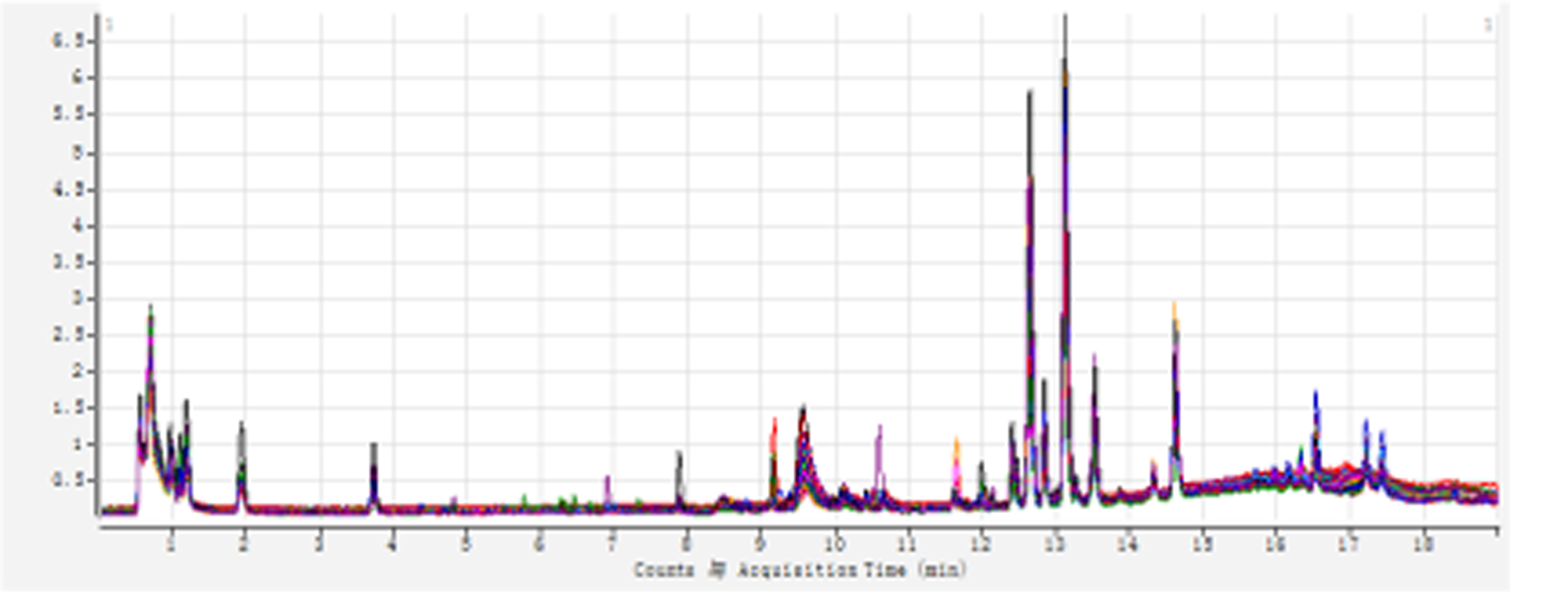

Supplement: S1 Fig — (TIF) [file pone.0126952.s001.tif]

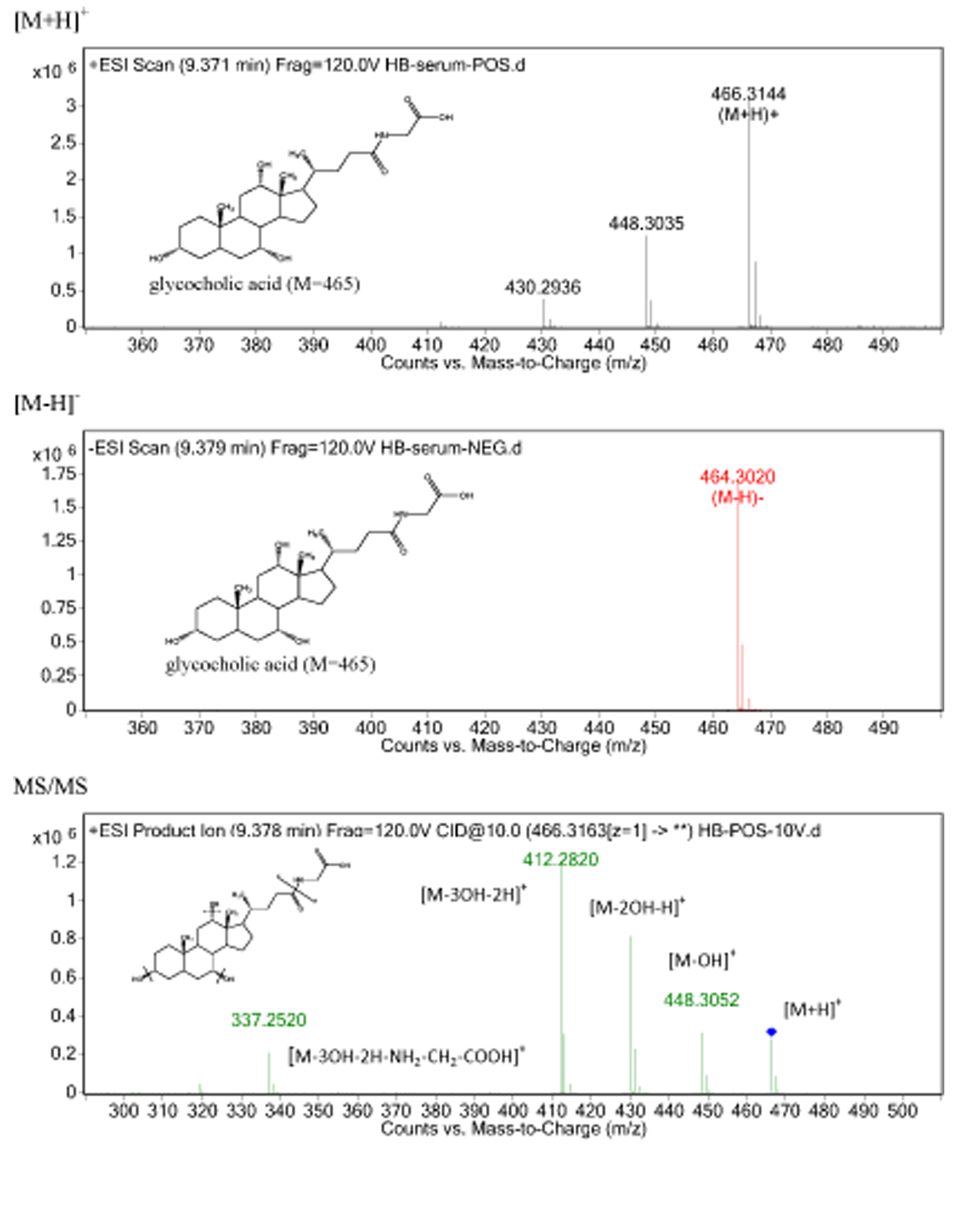

Supplement: S2 Fig — (TIF) [file pone.0126952.s002.tif]

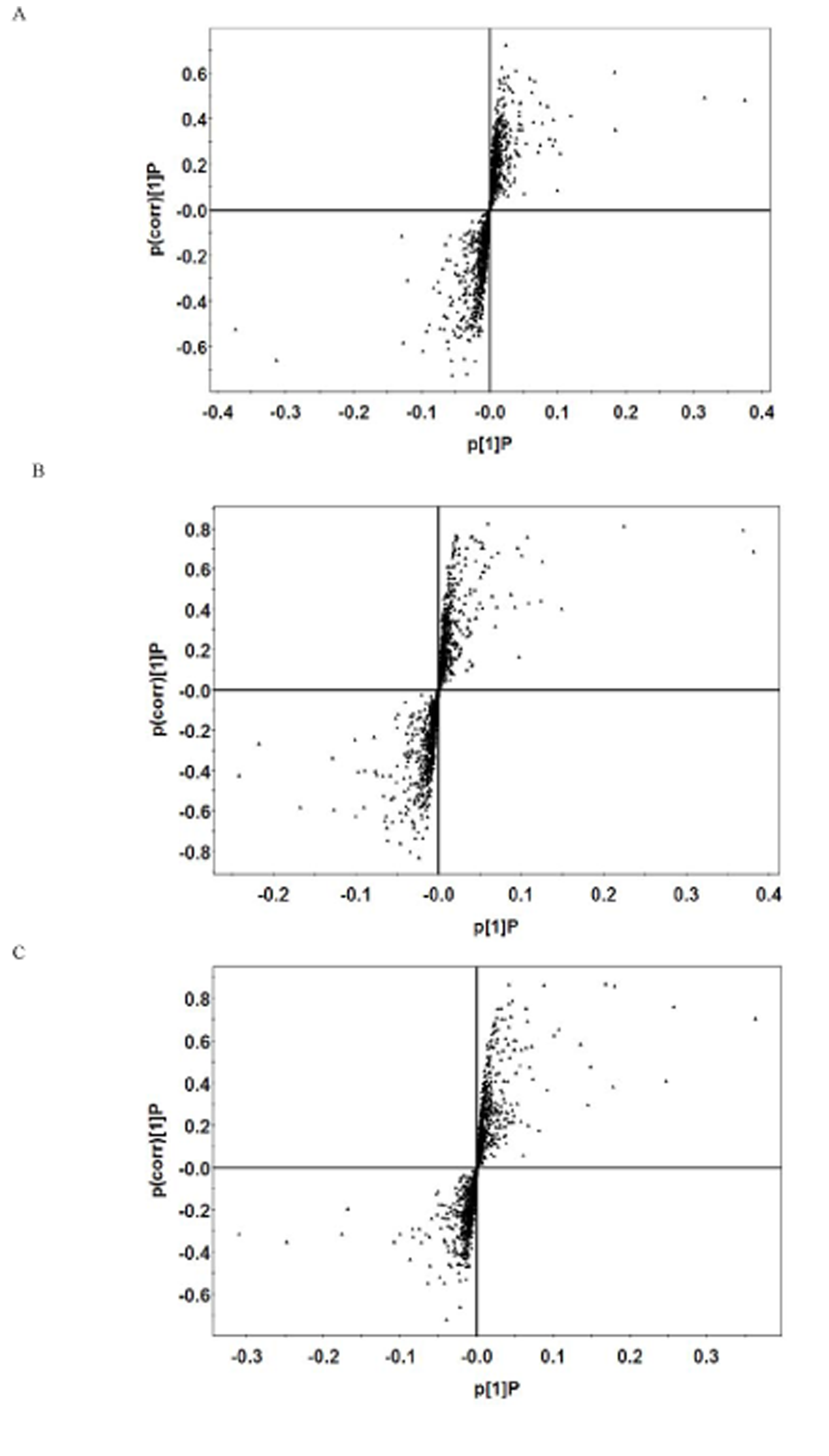

Supplement: S3 Fig — (A) T2DM vs Health control. (B) DACD vs Health control. (C) DACD vs T2DM. (TIF) [file pone.0126952.s003.tif]
