# Supplementary material for: Plasma Metabolomic Profiling of Patients with Diabetes-Associated Cognitive Decline
Source: PLoS One. 2015 May 14;10(5):e0126952. doi: 10.1371/journal.pone.0126952 (PMC4431856; doi:10.1371/journal.pone.0126952)
Supplement: S1 Table — (PDF) [file pone.0126952.s004.pdf]

S1 Table. Statistical analysis result of differential metabolites

Metabolites	Pathway	T2DM vs control				DACD vs control				DACD vs T2DM			
		vip[b]	p-value[c]	FDR	FC[d]	vip[b]	p-value[c]	FDR	FC[d]	vip[b]	p-value[c]	FDR	FC[d]
Phenylglyoxylic acid	Phenylalanine metabolism	1.606	0.014	0.027	-0.098	1.594	0.013	0.024	-0.087				
deoxyguanosine 5'-monophosphate (dGMP)	Purine metabolism	1.788	0.009	0.019	2.931	1.654	0.001	0.004	4.473	1.343	0.014	0.050	1.542
4-Nitroquinoline-1-oxide	others	1.565	0.007	0.017	-0.828	1.533	0.004	0.010	-0.897				
Nicotinate D-ribonucleotide	Nicotinate and nicotinamide metabolism	1.616	0.008	0.018	1.662	1.715	0.000	0.001	2.025				
Dimethyl fumarate	others	1.700	0.028	0.040	1.725								
Pipecolic acid	Lysine degradation	1.739	0.005	0.014	-1.335	1.536	0.001	0.005	-1.666				
Hypoxanthine	Purine metabolism	2.629	0.000	0.000	-0.598	2.051	0.000	0.000	-0.527				
Dopamine	Tyrosine metabolism	1.945	0.001	0.005	1.168	1.390	0.003	0.008	1.130				
L-Leucine	Valine, leucine and isoleucine degradation	1.253	0.038	0.050	0.250	1.434	0.023	0.038	0.348				
Indole-3-carboxylic acid	others	1.605	0.023	0.034	0.817	1.459	0.005	0.014	0.877				
3-Indoleacetic Acid	Tryptophan metabolism	1.861	0.003	0.012	3.259	1.157	0.029	0.041	3.001				
5'-Deoxy-5'-(methylthio)adenosine	others	1.784	0.004	0.013	2.702	1.786	0.000	0.001	3.502				
Hippuric acid	Phenylalanine metabolism	1.397	0.039	0.050	-1.293								
3-Indolebutyric acid	others	2.113	0.000	0.003	2.049	1.487	0.002	0.006	1.851				
Octanoylcarnitine	others	1.766	0.008	0.017	-0.640	1.457	0.024	0.038	-0.558				
PA(39:5)	others	2.060	0.003	0.012	1.640								
Decanoyl-L-carnitine	others	2.096	0.001	0.005	-0.767	1.639	0.006	0.014	-0.647				
PI(44:0)	others	1.792	0.006	0.016	0.860								

N-palmitoyl glutamic acid	others	1.255	0.009	0.019	-2.144								
Phytosphingosine	Sphingolipid metabolism	1.975	0.008	0.017	-0.837	3.104	0.000	0.000	1.627	4.310	0.000	0.000	2.508
25-hydroxy-cholesterol	Primary bile acid biosynthesis	2.205	0.002	0.009	-1.551								
N-stearoyl taurine	others	1.419	0.030	0.042	-1.124	1.642	0.007	0.016	-1.579				
N-palmitoyl histidine	others	1.406	0.017	0.029	-0.985								
N-Oleoyl-L-Serine	others	1.487	0.023	0.034	-0.685								
Sphinganine	Sphingolipid metabolism	2.106	0.003	0.012	-0.416	2.446	0.000	0.000	0.669	1.124	0.012	0.045	0.783
15(S)-15-methyl PGF2α ethyl amide	others	1.347	0.038	0.050	-0.916								
Glycerophospho-N-Arachido noyl Ethanolamine	others	1.624	0.020	0.031	0.307								
LysoPE(20:5)	others	1.808	0.005	0.016	0.367	1.115	0.037	0.047	1.412				
LysoPE(18:2)	others	1.428	0.027	0.039	0.328								
PC(18:2)	others	2.145	0.000	0.003	0.404	2.478	0.000	0.000	0.719	1.903	0.003	0.016	0.316
PC(22:6)	others	2.369	0.000	0.000	1.104	1.335	0.012	0.023	-1.444	3.219	0.000	0.000	1.283
Linoleyl carnitine	others	1.620	0.017	0.029	-0.329	1.721	0.001	0.004	-0.466				
N-docosanoyl taurine	others	2.193	0.001	0.005	-1.096								
PC(20:4)	others	1.815	0.006	0.016	0.882					2.703	0.000	0.000	0.856
Glycerophospho-N-Palmitoyl Ethanolamine	others	2.854	0.000	0.000	0.638	1.049	0.026	0.038	0.469	1.388	0.011	0.043	0.243
LysoPC(22:5)	others	1.714	0.005	0.015	0.541	2.370	0.000	0.000	1.576	2.534	0.000	0.001	1.002
LysoPE(18:3)	others	2.835	0.000	0.000	0.640	1.948	0.000	0.000	2.293	1.518	0.012	0.045	0.764
Palmitoyl-L-carnitine	Fatty acid degradation	1.445	0.040	0.050	0.185	1.281	0.009	0.018	0.307				
PC(16:0)	others	1.642	0.015	0.028	0.176	1.477	0.003	0.008	0.298				
LysoPC(18:3)	others	2.941	0.000	0.000	0.228	2.220	0.000	0.000	0.277	2.101	0.001	0.009	1.633
EPA	Biosynthesis of unsaturated fatty	2.053	0.003	0.012	0.697	1.712	0.001	0.003	0.756				

	acids												
PC(38:8)	others	1.785	0.003	0.012	1.698								
Adrenaline	Tyrosine metabolism					1.384	0.004	0.011	0.669				
Acetylcarnitine	others					1.160	0.017	0.031	-0.434	1.299	0.014	0.050	-0.444
Glycerophosphocholine	others					2.136	0.000	0.001	5.352	1.293	0.013	0.048	-1.564
Pyroglutamic acid	Glutathione metabolism					2.109	0.000	0.000	2.609	1.661	0.005	0.020	1.410
Phenylpyruvic acid	Phenylalanine metabolism					1.535	0.006	0.014	0.406	1.420	0.012	0.044	0.334
L-Phenylalanine	Cyanoamino acid metabolism					1.337	0.024	0.038	0.250				
Cystathionine	Biosynthesis of amino acids					1.747	0.001	0.004	0.536				
PGF1α	Arachidonic acid metabolism					1.022	0.027	0.040	1.453				
Taurocholic acid	Primary bile acid biosynthes					1.056	0.025	0.038	0.764	1.402	0.011	0.038	0.818
LysoPE(20:4)	others					1.687	0.001	0.003	0.482				
Taurodeoxycholic acid	others					1.174	0.020	0.035	0.322				
LysoPE(22:6)	others					1.465	0.002	0.006	0.371				
LysoPC(22:6)	others					1.629	0.001	0.003	1.240				
PE(16:0)	others					2.415	0.000	0.000	0.824				
α-Linolenoyl Ethanolamide	others					1.571	0.006	0.014	0.174				
Arachidonoyl dopamine	Primary bile acid biosynthesis					1.646	0.001	0.003	2.229				
LysoPE(18:1)	others					1.089	0.032	0.041	0.318				
PC(18:1)	others					1.752	0.000	0.001	0.522				
Arachidyl carnitine	others					1.276	0.029	0.041	-1.050				
PGF2α Alcohol methyl ether	others					1.094	0.023	0.038	1.701	1.562	0.010	0.032	1.738
Nα-Acetyl-L-glutamine	others									1.350	0.013	0.049	-1.053
Phosphocholine	Ether lipid metabolism									1.672	0.012	0.043	1.464
LysoPC(20:3)	others									2.867	0.000	0.001	0.921
LysoPE(22:1)	others									1.410	0.013	0.048	1.231

LysoPE(20:3)	others									1.600	0.010	0.035	1.657
Cholic acid	Primary bile acid biosynthesis	2.249	0.007	0.017	2.730					2.117	0.011	0.038	-3.003
Cinnamic acid	Phenylpropanoid biosynthesis	2.124	0.014	0.027	-2.953								
corticosterone	others					1.671	0.040	0.050	-1.317				
Deoxycholic acid	Primary bile acid biosynthesis	2.381	0.003	0.012	3.445	1.132	0.031	0.042	0.669	2.087	0.013	0.047	-2.668
D-Glucose 6-sulfate	others	1.825	0.041	0.050	-0.903								
Glucoheptonic acid	others	2.659	0.000	0.003	0.534								
Glycocholic Acid	Primary bile acid biosynthesis					1.201	0.023	0.038	2.167	2.070	0.002	0.011	0.685
Indoxylsulfuric acid	others	1.838	0.040	0.050	-1.421								
Lipoxin C4	others					1.615	0.040	0.050	-0.726	1.786	0.014	0.050	-1.067
PC(17:0)	others	1.855	0.039	0.049	-0.311								
PC(18:3)	others					2.039	0.000	0.000	3.111				
PC(19:0)	others	2.041	0.020	0.031	-0.866								
PE(20:3)	others									2.340	0.003	0.016	1.571
Sphinganine-phosphate	sphingolipid metabolism									2.122	0.011	0.038	1.530
Sphingosine-1-phosphate	sphingolipid metabolism	2.135	0.013	0.026	-0.277								
Uric acid	Purine metabolism	2.033	0.020	0.031	4.338								
α-D-Glucose/D-Fructose	Glycolysis / Gluconeogenesis	2.071	0.017	0.029	0.704	1.805	0.031	0.042	0.648				
γ-Linolenic Acid/α-Linolenic Acid	Linoleic acid metabolism	2.082	0.017	0.029	1.497								
2-keto valeric acid	others					2.044	0.011	0.022	-1.510	2.137	0.010	0.038	-1.620
3-Methylxanthine/7-Methylxanthine/1-Methylxanthine	Caffeine metabolism					1.922	0.019	0.034	1.905	1.886	0.012	0.042	2.143
Arachidonic Acid	Arachidonic acid metabolism					1.646	0.039	0.049	-0.711				
Glycerophospho-N-Oleoyl Ethanolamine	others	1.325	0.041	0.050	0.409	1.918	0.000	0.001	0.689				

LysoPE(22:5)	others					1.189	0.014	0.026	0.553	1.946	0.003	0.016	0.584
PC(14:0)	others					2.432	0.001	0.018	1.316	1.491	0.014	0.050	0.446
PC(15:0)	others	2.439	0.000	0.003	0.368	2.345	0.002	0.006	0.545	1.835	0.013	0.046	0.447
PE(16:1)	others					1.701	0.040	0.050	0.753				
PE(18:2)	others					1.820	0.029	0.041	0.633				
PE(18:3)	others					1.795	0.032	0.042	1.281				
Uridine monophosphate	Peptidoglycan biosynthesis					1.636	0.038	0.049	-1.513	1.861	0.011	0.037	-1.727
Xanthine	Caffeine metabolism					1.627	0.039	0.050	-1.040				

[a]Abbreviations: T2DM, Health control, and DACD. [b]Variable importance in the projection (VIP) was obtained from PLS-DA with a threshold of 1.0. [c]The p-value and FDR-value were calculated from the nonparametric Kruskal−Wallis rank sum test. [d]Fold change (FC) was calculated from the mean values of each group.
