# Supplementary material for: Plasma Metabolomic Profiling of Patients with Diabetes-Associated Cognitive Decline
Source: PLoS One. 2015 May 14;10(5):e0126952. doi: 10.1371/journal.pone.0126952 (PMC4431856; doi:10.1371/journal.pone.0126952)
Supplement: S2 Table — (PDF) [file pone.0126952.s005.pdf]

S2 Table. The informations of reference standards

Name	Formula	Mass	M+H	M+Na	M-H	M+FA-H	Rt(Standard)	Rt(sample)
Glycerophosphocholine	$C_8H_{20}NO_6P$	257.1028	258.1106	280.092	256.0955	302.1014	0.70	0.70
phytosphingosine	$C_{18}H_{39}NO_3$	317.293	318.3003	340.2822	316.2857	362.2912	11.15	11.13
Glycocholic acid	$C_{26}H_{43}NO_6$	465.309	466.3163	488.2983	464.3018	510.3072	9.37	9.37
Sphingosine-1-phosphate	$C_{18}H_{38}NO_5P$	379.2488	380.256	402.238	378.2415	—	11.60	11.60
sphinganine-phosphate	$C_{18}H_{40}NO_5P$	381.2644	382.2717	404.2536	380.2571	426.2626	11.87	11.87
Pyroglutamic acid	$C_5H_7NO_3$	129.0426	130.0499	152.0318	128.0353	174.0408	0.75	0.75
Hypoxanthine	$C_5H_4N_4O$	136.0385	137.0458	159.0277	135.0312	181.0367	1.08	1.08
Cholic acid	$C_{24}H_{40}O_5$	408.2876	—	—	407.2803	453.2858	10.52	10.52
Linoleic acid	$C_{18}H_{32}O_2$	280.2402	281.2475	303.2295	279.233	325.2384	16.60	16.60
