# Supplementary material for: Plasma Metabolomic Profiling of Patients with Diabetes-Associated Cognitive Decline
Source: PLoS One. 2015 May 14;10(5):e0126952. doi: 10.1371/journal.pone.0126952 (PMC4431856; doi:10.1371/journal.pone.0126952)
Supplement: S3 Table — (PDF) [file pone.0126952.s006.pdf]

S3 Table. the detailed results from the pathway analysis

| **Pathway** | **Matched Entities** | **Pathway Entities of Experiment Type** | **Raw *P* value** |
|---|---|---|---|
| Hs_Selenium_Pathway_WP15_56489 | 8 | 105 | <0.001 |
| Hs_Sphingolipid_Metabolism_WP1923_46972 | 4 | 29 | <0.0007 |
| Hs_Transport_of_glucose_and_other_sugars,_bile_salts_and_organic_acids,_metal_ions_and_amine_compounds_WP1935_45063 | 3 | 29 | <0.02 |
| Hs_Glucose_Homeostasis_WP661_45308 | 3 | 21 | <0.004 |
| Hs_Drug_Induction_of_Bile_Acid_Pathway_WP2289_57150 | 3 | 9 | <0.01 |
| Hs_Metabolism_of_amino_acids_and_derivatives_WP1847_52373 | 4 | 135 | <0.005 |
| purine nucleotides degradation II (aerobic) | 3 | 19 | <0.0004 |
| Hs_Glucose_Homeostasis_WP661_34501 | 3 | 21 | <0.001 |
| Hs_Metabolism_of_nucleotides_WP1851_44898 | 3 | 81 | <0.01 |
| adenosine nucleotides degradation II | 3 | 13 | <0.003 |
| Hs_Glucose_Homeostasis_WP661_45308 | 3 | 21 | <0.004 |
| Hs_Phase_1_-_Functionalization_of_compounds_WP1879_42095 | 3 | 102 | <0.01 |
| Hs_Drug_Induction_of_Bile_Acid_Pathway_WP2289_57150 | 3 | 9 | <0.002 |
| Hs_Nucleotide_Metabolism_WP404_45328 | 3 | 17 | <0.01 |
| Hs_Transport_of_glucose_and_other_sugars,_bile_salts_and_organic_acids,_metal_ions_and_amine_compounds_WP1935_45063 | 3 | 29 | <0.02 |

Many pathways were tested at the same time, Raw *P* the original *P* value calculated from the entichment analysis showing the P value of the changes in the pathway.
